# Supplementary material for: Deciphering the CircRNA-Regulated Response of Western Honey Bee (Apis mellifera) Workers to Microsporidian Invasion
Source: Biology (Basel). 2022 Aug 29;11(9):1285. doi: 10.3390/biology11091285 (PMC9495892; doi:10.3390/biology11091285)
Supplement: Supplementary file 1 [file biology-11-01285-s001.zip › Supplementary Figure S1.pdf]

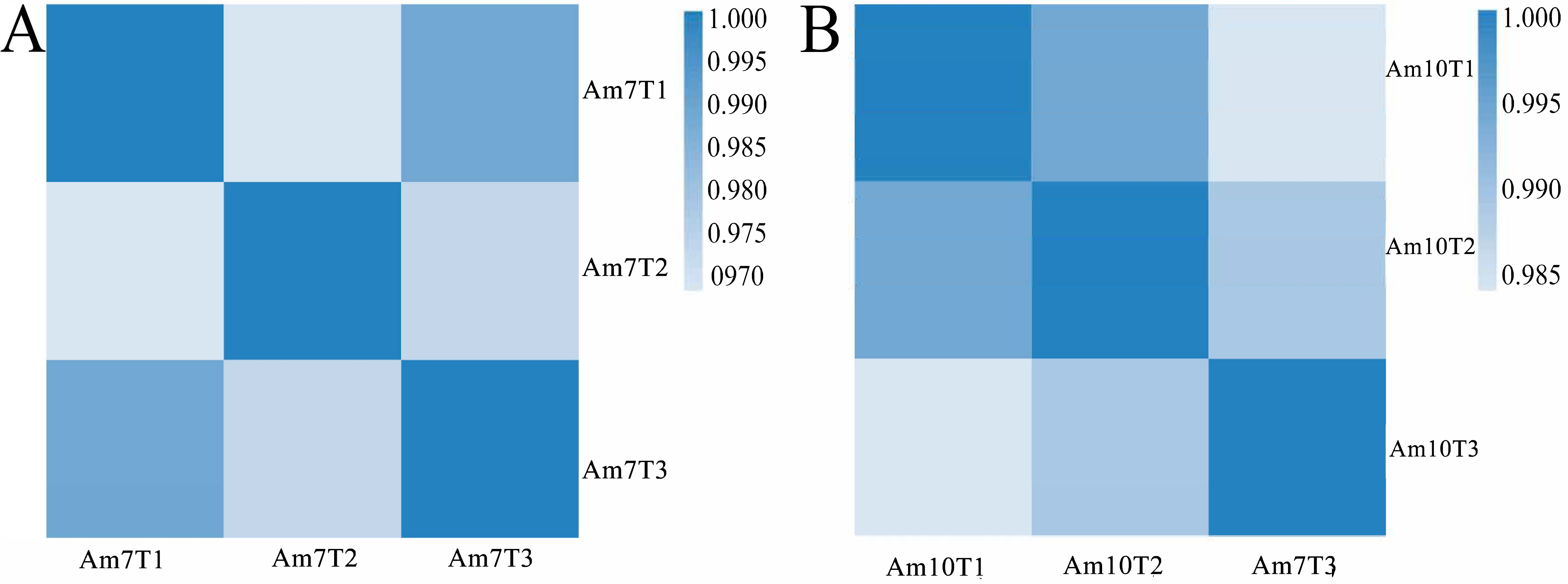

Figure S1. Pearson correlation coefficient between different biological repeats of midgut samples of *A. m. ligustica* workers. (**A**) Pearson correlation coefficient between three biological repeats of midgut samples of *V. ceranae* at 7 dpi in *A. m. ligustica* workers. (**B**) Pearson correlation coefficient between three biological repeats of midgut samples of *V. ceranae* at 10 dpi in *A. m. ligustica* workers.
