# Supplementary material for: Deciphering the CircRNA-Regulated Response of Western Honey Bee (Apis mellifera) Workers to Microsporidian Invasion
Source: Biology (Basel). 2022 Aug 29;11(9):1285. doi: 10.3390/biology11091285 (PMC9495892; doi:10.3390/biology11091285)
Supplement: Supplementary file 1 [file biology-11-01285-s001.zip › Supplementary Table S1.pdf]

Supplementary Table S1 Divergent primers and convergent primer of molecular validation of novel circRNAs and DEcircRNAs, primers for Stem-loop PCR validation of circRNA target miRNAs

Id	Type of primers	Sequence	Product size (bp)	Purpose
Novel_circ_004065	Divergent Primer	F: CTCCACAATGTTTACCTGGTC<br>R: ATCTCCGAGTTCTTGAGCG	86	
	Convergent primer	F: TCCTCATTCCCTCATCAATC<br>R: CGCTTTCAGCAGATACTCG	301	
Novel_circ_002199	Divergent Primer	F: AACAGCGTTGAATCAGGC<br>R: TTCGGGCAAAGGATGTAG	187	RT-PCR of novel circRNAs
	Convergent primer	F: GGAAGAAGAAGCGAGCAAG<br>R: TACAATGGGAGAGTCAGTGG	358	
Novel_circ_005784	Divergent Primer	F: ACACCTGCTGCTACACCATC<br>R:GCTGGAGTTACTGCGTATCC	91	
	Convergent primer	F: CATACGATTACGGTTACGGAC<br>R: TTGTCGCTGTTGATGGTG	312	
Novel_circ_000705	Divergent Primer	F: CAAGTCCAAGGCGAAGAAG<br>R: CCCTCGGTGTTCAACTGTAG	259	
	Convergent primer	F: ATCTTCTGGACACCATCAGC<br>R: TCTCGTCTTTGGATTCTCG	264	
Novel_circ_001195	Divergent Primer	F: ATAATGGCGGTTCGCTGAG<br>R: ATTGCCTAACAAAGTTGGAGGG	220	
	Convergent primer	F: TCTCGCATTGTTCTCAGGG<br>R: AAGCGGTTTCCTCTCGTCAC	351	
Novel_circ_011173	Divergent Primer	F: AGAGCGTGGAAAGCAGAAC<br>R: GGAAAGAGAAAGAATGGTCG	255	RT-qPCR of DEcircRNAs
	Convergent primer	F: GGAGAAAGAAAGAATGCGTG<br>R: GCCTGCTTGAAGATTTGC	455	
Novel_circ_006925	Divergent Primer	F: CCCTTATCCGTTGGGTATG<br>R: ACAAAGAGGCGTGGAAAC	244	
	Convergent primer	F: TCCACGCCTCTTTGTATCC<br>R:GCAGTCTCTGACGATGGTAAG	290	
Novel_circ_012352	Divergent Primer	F: GCTACCGTATTGCCATTCAC<br>R: ACATTGATGCTGGTGTCG	133	
	Convergent	F: ACGAAACGACGACGAGTTC<br>R: TGAATGGCAATACGGTAGC	344	

	primer	R: GTGATTGCTGTTGTCGTTG		
Novel_circ_012316	Divergent Primer	F: CCTGTCTCTCCACAAATGTTTC<br>R: TGTTCTCACGGTTACGGAC	244	
	Convergent primer	F: CTATGCCACCCATTCCTAAC<br>R: ATGTAAACGGCGGTCTGAC	324	
Novel_circ_007686	Divergent Primer	F: AACAATACACTTGCCCAGG<br>R: TCTTCCGCCAATCTGAAC	131	
	Convergent primer	F: TCGGTTTCGTTAGGGAGAC<br>R: TGGACACCAGATGATTCG	312	
Novel_circ_011500	Divergent Primer	F: GCAATCCAAGGACAATCTG<br>R: TATTCCAGTCTGTGGGCTG	166	
	Convergent primer	F: AACAGCATCAGCAACAAGC<br>R: GGATTTCCGTTCCTAAGTGC	130	
actin		F: CACTCCTGCTATGTATGTCGC<br>R: GGCAAAGCGTATCCTTCA	132	
Ame-mir-3720-loop		5'-CTCAACTGGTGTCGTGGAGTCGGCAATTCAGTTGAGCTGTTTAA-3'		
Mir-21-x-loop		5'-CTCAACTGGTGTCGTGGAGTCGGCAATTCAGTTGAGGTCAACAT-3'		
Mir-30-x-loop		5'-CTCAACTGGTGTCGTGGAGTCGGCAATTCAGTTGAGAGCTTCCA-3'		
Mir-29-y-loop		5'-CTCAACTGGTGTCGTGGAGTCGGCAATTCAGTTGAGTAACCGAT-3'		
Mir-451-x-loop	/	5'-CTCAACTGGTGTCGTGGAGTCGGCAATTCAGTTGAGACTCAGTA-3'	/	Stem-loop PCR of circRNA target miRNAs
Mir-7975-y-loop		5'-CTCAACTGGTGTCGTGGAGTCGGCAATTCAGTTGAGTGGTGCCG-3'		
Mir-146-x-loop		5'-CTCAACTGGTGTCGTGGAGTCGGCAATTCAGTTGAGCCATCTAT-3'		
Mir-143-y-loop		5'-CTCAACTGGTGTCGTGGAGTCGGCAATTCAGTTGAGGAGCTACA-3'		
Mir-101-y-loop		5'-CTCAACTGGTGTCGTGGAGTCGGCAATTCAGTTGAGCTTCAGTT-		

	3'
Mir-462-x-loop	5'-CTCAACTGGTGTCGTGGAGTCGGCAATTCAGTTGAGCAGCTGCA-3'
Ame-mir-3720-F	5'-ACACTCCAGCTGGGATACGGTGATGAGT-3'
Mir-21-x-F	5'-ACACTCCAGCTGGGTAGCTTATCAGACTG-3'
Mir-30-x-F	5'-ACACTCCAGCTGGGTGTAAACATCCTCGAC-3'
Mir-29-y-F	5'-ACACTCCAGCTGGGTAGCACCATCTGAA-3'
Mir-451-x-F	5'-ACACTCCAGCTGGGAAACCGTTACCAT-3'
Mir-7975-y-F	5'-ACACTCCAGCTGGGATCCTGGTCA-3'
Mir-146-x-F	5'-ACACTCCAGCTGGGTGAGAACTGAATTCC-3'
Mir-143-y-F	5'-ACACTCCAGCTGGGTGAGATGAAGCAC-3'
Mir-101-y-F	5'-ACACTCCAGCTGGGTACAGTACTGTGAT-3'
Mir-462-x-F	5'-ACACTCCAGCTGGGTAACGGAACCCATAA-3'
Universal R	5'-CTCAACTGGTGTCGTGGA-3'
